# Potential role of autophagy induced by FLT3-ITD and acid ceramidase in acute myeloid leukemia chemo-resistance: new insights

**DOI:** 10.1186/s12964-022-00956-7

**Published:** 2022-10-31

**Authors:** Hamidreza Zalpoor, Maryam Bakhtiyari, Abdullatif Akbari, Fatemeh Aziziyan, Hooriyeh Shapourian, Mahsa Liaghat, Zahra Zare-Badie, Sheida Yahyazadeh, Vahideh Tarhriz, Mazdak Ganjalikhani-Hakemi

**Affiliations:** 1 Shiraz Neuroscience Research Center, Shiraz University of Medical Sciences, Shiraz, Iran; 2 Network of Immunity in Infection, Malignancy and Autoimmunity (NIIMA), Universal Scientific Education and Research Network (USERN), Tehran, Iran; 3 Department of Medical Laboratory Sciences, Faculty of Allied Medicine, Qazvin University of Medical Sciences, Qazvin, Iran; 4 Department of Biochemistry, Faculty of Biological Sciences, Tarbiat Modares University, Tehran, Iran; 5 Department of Immunology, Faculty of Medicine, Isfahan University of Medical Sciences, Isfahan, Iran; 6 Department of Medical Laboratory Sciences, Faculty of Medical Sciences, Kazerun Branch, Islamic Azad University, Kazerun, Iran; 7 Diagnostic Laboratory Sciences and Technology Research Center, School of Paramedical Sciences, Shiraz University of Medical Sciences, Shiraz, Iran; 8 Molecular Medicine Research Center, Biomedicine Institute, Tabriz University of Medical Sciences, Tabriz, Iran

**Keywords:** Acute myeloid leukemia, FLT3-ITD, Autophagy, Acid ceramidase, Sphingosine, Chemo-resistance

## Abstract

Acute myeloid leukemia (AML) is a type of leukemia with a poor prognosis and survival characterized by abnormal cell proliferation and differentiation. Despite advances in treatment, AML still has a low complete remission rate, particularly in elderly patients, and recurrences are frequently seen even after complete remissions. The major challenge in treating AML is the resistance of leukemia cells to chemotherapy drugs. Thus, to overcome this issue, it can be crucial to conduct new investigations to explore the mechanisms of chemo-resistance in AML and target them. In this review, the potential role of autophagy induced by FLT3-ITD and acid ceramidase in chemo-resistance in AML patients are analyzed. With regard to the high prevalence of FLT3-ITD mutation (about 25% of AML cases) and high level of acid ceramidase in these patients, we hypothesized that both of these factors could lead to chemo-resistance by inducing autophagy. Therefore, pharmacological targeting of autophagy, FLT3-ITD, and acid ceramidase production could be a promising therapeutic approach for such AML patients to overcome chemo-resistance.

**Video abstract**

**Supplementary Information:**

The online version contains supplementary material available at 10.1186/s12964-022-00956-7.

## Introduction

### Chemo-resistance in AML and its challenges

Acute myeloid leukemia (AML) is the most common acute leukemia in adults [1]. It is characterized by clonal proliferation of undifferentiated myeloid precursors, thereby impairing hematopoiesis and giving rise to bone marrow failure [2]. Several genetic mutations provide diagnostic and prognostic data, including mutations in FMS-like tyrosine kinase-3 (FLT3), Nucleophosmin 1 (NPM1), KIT, CCAAT/enhancer-binding protein alpha (CEBPA), and ten-eleven-translocation 2 (TET2) [3]. While many patients respond well to induction chemotherapy, chemo-resistance is a significant challenge in the treatment of AML patients particularly older patients are more resistant to treatment making the cure more challenging and only 5–25% of them are likely to achieve a cure [4]. Therefore, drug resistance is a major barrier to cancer chemotherapy [5]. Some gene mutations like FLT3, autophagy signal pathway, drug resistance-related protein and enzyme-like MDR (multiple drug resistance), and abnormal expression of microRNA (miRNA) will cause drug resistance in AML patients [6]. Although patients undergoing initial induction chemotherapy can attain a complete response rate as high as 80%, most AML patients will eventually be diagnosed with recurrent or refractory disease [6]. A therapeutic strategy for AML patients is an intensive induction regimen including Cytarabine and Anthracycline infusion that is usually used to induce complete remission. A complete remission is usually achieved in about 60–80% of younger adults and 40–60% older than 65 years. In addition, allogeneic stem cell transplants (alloSCT) are recommended for AML patients with poor prognosis, and the post-remission regimen consists of consolidation chemotherapy, autologous and allogeneic stem cell transplantation [7].

### FLT3-ITD and acid ceramidase levels and related association with drug resistance in AML patients

Around 25% of AML cases have mutations in the FLT3 receptor [8]. FLT3 is an oncogene in AML, which exhibits two types of mutations; internal tandem duplication (ITD) in 20–25% of patients, and tyrosine kinase domain (TKD) mutations in 5–10% of patients [6, 9]. FLT3 is a tyrosine kinase receptor that contributes to the differentiation, proliferation, and apoptosis of hematopoietic stem cells [7]. Mutation in FLT3-ITD has been identified as a relapse-associated genetic marker [6]. Myelodysplastic syndromes (MDS) and AML are hematological malignancies associated with aberrant splicing patterns [10] and FLT3 is one of the most mis-spliced genes in AML, dysregulation of the splicing process can also disturb apoptosis and cause resistance to therapies [4]. FLT3 mutation carriers, especially those with ITD mutations, are more likely to have poor prognoses, with fewer chances of complete remission and overall survival [11].

Furthermore, in AML, acid ceramide expression is elevated. Overexpression of acid ceramidase increases ceramide breakdown and increases S1P production. In healthy cells, a stable balance exists between ceramides and S1Ps levels, but disruption of this equilibrium contributes to the progression of several diseases including multiple cancers [12]. Thus, there is an elevated level of acid ceramidase in several types of solid tumors and leukemia, such as AML, and patients with elevated acid ceramidase have a poor prognosis and lower overall survival [13]. As ceramide has a pro-apoptotic role and sphingosine has a pro-survival role, acid ceramidase promotes cellular proliferation and increases the growth rate of tumor cells. In addition, upregulation of acid ceramidase contributes to chemo-resistance in solid tumors such as prostate cancer and hepatoma cancer cell lines [14]. It is also shown that a high acid ceramidase level can increase the survival of AML cells [12].

### Autophagy and drug resistance in AML and other malignancies

In recent years, there have been an increase in autophagy research. By definition, autophagy is the process of phagocytosing its own cytoplasmic organelles or proteins. Autophagy originates from a Greek word that means eating oneself. During autophagy, old proteins or organelles are broken down by lysosomes within the cells, nutrients and new synthetic materials are supplied to maintain cellular homeostasis [15, 16]. As its package is transferred into vesicles, it will be fused with lysosomes, creating autolysosomes, and eventually degrading its contents. During this process, organelles and cell metabolism are renewed. A chemotherapeutic drug can induce autophagy, resulting in drug resistance in cancer cells [6]. A study by Shang et al. has shown that cirpan3 (a kind of circular RNA) is involved in drug resistance in AML by promoting autophagy through the AMPK/mTOR pathway [17]. In other leukemias, such as chronic lymphocytic leukemia (CLL), bone marrow stromal cells stimulate autophagy which contributes to drug resistance in CLL cells [18]. As well, autophagy has a pro-survival role in Multiple Myeloma (MM) and it promotes the resistance of MM cells to proteasome inhibitors [19]. Moreover, autophagy can lead to chemo-resistance in solid tumors such as prostate and renal cancer cells [20]. In glioblastoma, hypoxia induces autophagy, which contributes to tumor cell survival and resistance to antiangiogenic treatments [21]. Therefore, autophagy is a significant process that can lead to chemo-resistance in AML and other malignancies [22–24]. This suggests that it is a potential target in cancer treatments for preventing or reducing chemo-resistance.

## The role of FLT3-ITD in induction of autophagy

Based on studies in xenografted mice, FLT3-ITD activity increased basal autophagy in AML cells, which is needed for cell proliferation in vitro. FLT3-ITD-induced autophagy was found to be mediated by ATF4 (activating transcription factor 4). Downregulation of autophagy and ATF4 inhibits the proliferation of AML cells and improves survival in mice. It appears that autophagy is involved in the proliferation and degradation of the FLT3-ITD receptor [25]. The RET receptor, a tyrosine kinase receptor often activated in AML, has been demonstrated to suppress autophagy via mTORC1 (mammalian target of rapamycin complex1), leading to the stabilization of mutant FLT3 receptors [26].

Recent studies have found that SHP2, which interacts with FLT3-ITD phosphorylated Gab2, is involved in activating the MEK/ERK pathway, as well as RSK's negative feedback regulation of that pathway in FLT3-ITD-positive AML cells. Activation of the MEK/ERK or phosphatidylinositol 3-kinase (PI3K)/AKT pathways can occur through the interaction of SHP2 or p85 with tyrosine-phosphorylated Gab2 via their SH2 domains, respectively, downstream of several tyrosine kinases or cytokine receptors [27, 28]. FLT3-ITD is capable of triggering the transcription factors signal transducer and activator of transcription (STAT) 5. STAT5 to translocate into the nucleus, triggering the production of oncogenic proteins such as (proviral insertion site) PIM kinases and Bcl-xL [29]. Through the STAT5/PIM and PI3K/AKT pathways, FLT3-ITD cooperatively activates the mTORC1/S6K/4EBP1 pathway [30].

By activating mTORC1, several cellular processes can be regulated that affect the metabolic state of the cell. A number of mechanisms underlie mTORC1 signaling, including inhibition of autophagy and stimulation of biosynthesis pathways [31].

The mTORC1 pathway inhibits autophagy at a number of stages. The mechanism through which mTORC1 inhibits autophagy is best understood as direct control of unc-51-like autophagy activating kinase 1 (ULK1), but it is also associated with the human class III PI3K (Vps34) complex containing autophagy-related protein 14 (ATG14) and transcription factor EB (TFEB). The autophagy initiators ULK1 and ATG13, which form a complex with the focal adhesion kinase family interacting protein of 200KDa (FIP200) and ATG101, are inactivated by mTORC1 in amino acid-rich conditions which binds, phosphorylates, and thus inactivates ULK1 [31–33].

A second mechanism involves mTORC1 phosphorylating TFEB and TFE3 and this event allows them to interact with the cytosolic chaperone 14-3-3 to remain in the cytoplasm [34, 35].

Lack of activating stimuli, in turn, induces autophagy through dissociation between mTORC1 and the ULK1 complex, thereby reducing the inhibition of ULK1, which is then phosphorylated along with ATG13, FIP200, and Raptor [33].

**Fig. 1:**
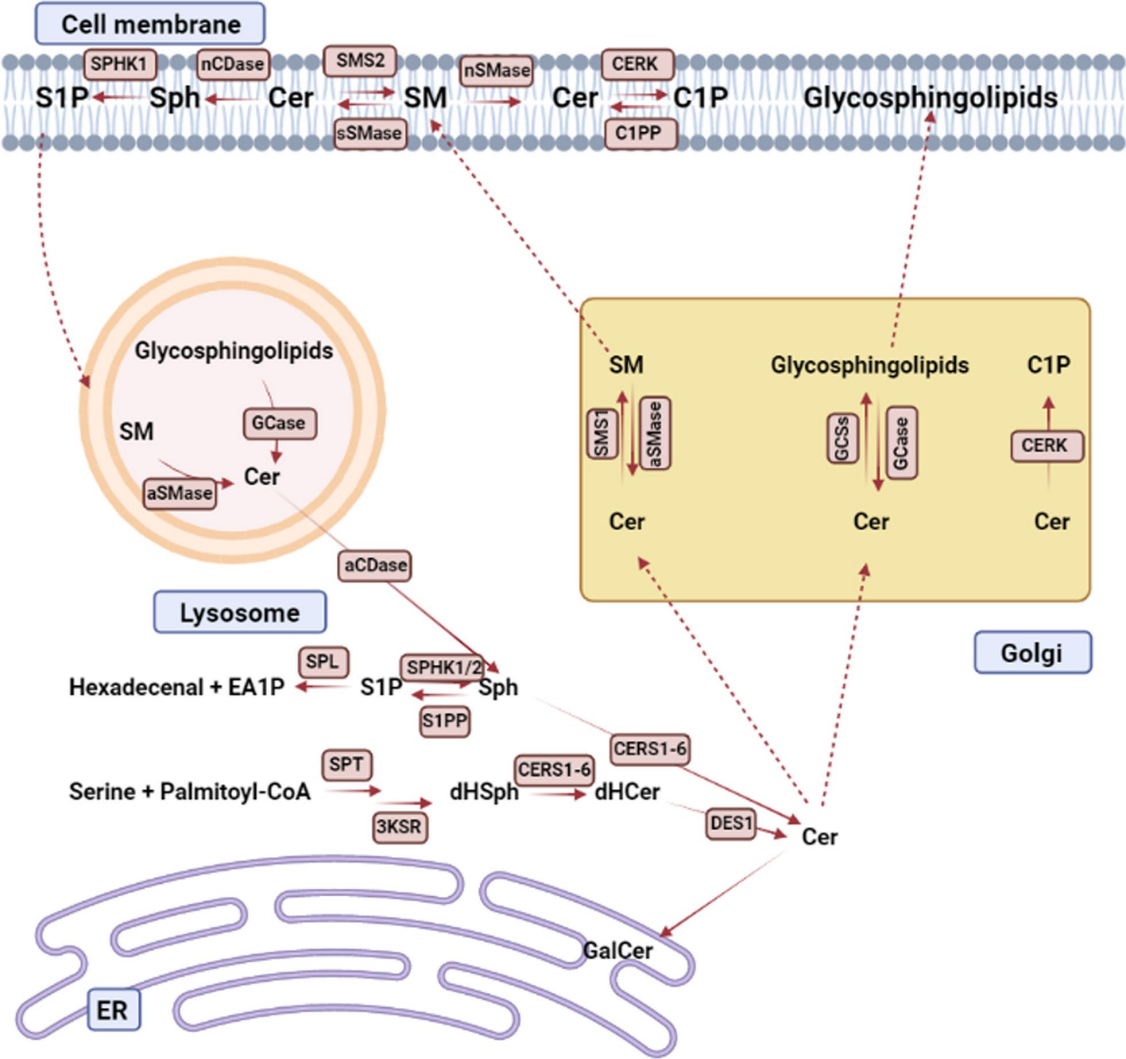
Sphingolipid metabolism in a summary. Ceramide is a key component of sphingolipid metabolism. Ceramide is made from the condensation of serine and palmitoyl-CoA in the endoplasmatic reticulum (ER) by serine palmitoyltransferase (SPT). After further reduction and acylation by a ceramide synthase (CERS1-6), dihydrosphingosine (dHSph) is produced, which is then desaturated to generate ceramide. Ceramide can be altered to galactosylceramide (GalCer) in the ER, although most ceramide change occurs at the Golgi in a manner that is dependent on their following use. Ceramide is used in the Golgi to produce sphingomyelin (SM) and glycosphingolipids in processes mediated by sphingomyelin synthase 1 (SMS1) and glycosphingolipid synthases (GCSs). SM and glycosphingolipids are delivered to the plasma membrane from the Golgi. By the actions of secretory and neutral sphingomyelinases, SM can be converted back to ceramide (sSMase and nSMase). Ceramide can then be converted to ceramide-1-phosphate (C1P), sphingosine-1-phosphate (S1P), or SM. By accessing the endolysosomal process, complex sphingolipids in the membrane can be utilized as a pool for ceramide recycling. Acid SMases (aSMase) and glycosidases (GCase) create ceramide, which can then be hydrolyzed into sphingosine and utilized in ceramide production or degraded by phosphorylation into S1P followed by degradation to hexadecenal and ethanolamine-1-phosphate (EA1P). Ceramide kinase (CERK) can phosphorylate ceramide in the Golgi, resulting in ceramide-1-phosphate (C1P)

ULK1 can then activate the PI3K complex and induce autophagosome synthesis. Furthermore, mTORC1 inactivation leads to the re-localization of TFEB and TFE3 to the nucleus, where they induce the expression of multiple autophagy-related genes. As a result, the cell maintains a critical level of energy and metabolites in order to survive the starvation state (Fig. 1) [36].

**Fig. 2:**
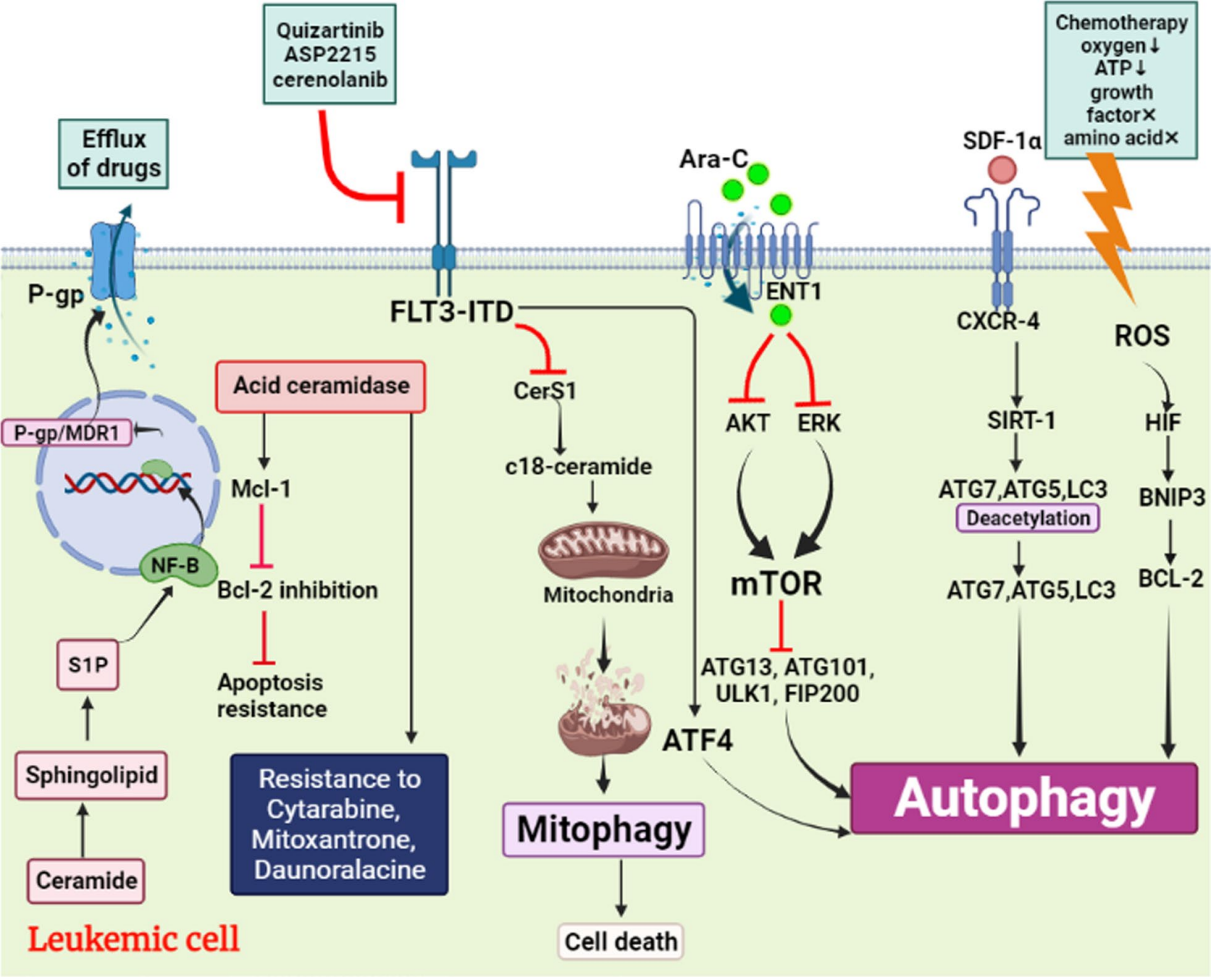
Illustration shows that leukemic cells design mechanisms against a variety of therapies and ultimately achieve chemotherapy-resistance through autophagy: 1—Chemotherapy increases reactive oxygen species (ROS) and induces autophagy through HIF/ BNIP3/BCL-2 signaling pathway. 2—Binding of SDF-1α as a drug to CXCR-4 activates SIRT-1 and SIRT-1 by deacetylation of ATG7, ATG5, LC3 induces autophagy. 3—Ara-C modulates AKT and ERK signaling pathways and induces mTOR-dependent autophagy. 4—By activating ATF4, it directly induces autophagy and inhibits mitophagy by inhibiting CerS1. 5—S1P production as a result of increased ceramidase acid in AML cells activates NF-κB and increases P-gp expression as a channel that causes drug efflux

Nevertheless, these signaling pathways connected to mTOR and ULK1 are controversial, and it is also possible for FLT3-ITD to promote autophagy through some other mechanisms and signaling pathways. According to the above evidence, for the first time, in a study by Heydt et al. [25], ATF4 (a transcription factor) was recognized as a critical factor of FLT3-ITD-induced autophagy (Fig. 2). The level of ATF4 in cells was highly dependent on FLT3-ITD activity, and ATF4 downregulation inhibited autophagy and cell proliferation in AML cells and increased survival of mice in a similar manner to autophagy inhibition.

Based on available evidence, targeting autophagy or ATF4 in patients expressing FLT3 mutations could be a novel potential promising therapeutic approach for AML patients with or even without significant chemo-resistance. In order to provide other meaningful insights into chemo-resistance in AML patients, more studies are needed to figure out the exact mechanism of correlations between FLT3-ITD and autophagy.

## The role of acid ceramidase and ceramide in induction of autophagy

Acid ceramidase is a hydrolase that converts ceramide into sphingosine and free fatty acids, located in the lysosome. By degrading ceramide into its metabolites, ceramide decreases in the cell while sphingosine increases [37]. Sphingosine is then phosphorylated and converted into sphingosine-1-phosphate (S1P) by sphingosine kinase [38]. S1P and ceramide play roles in determining whether cells undergo apoptosis or proliferation [39]. The accumulation of ceramide in the cell causes apoptosis. In contrast, S1P stimulates angiogenesis through G protein-coupled receptors [37]. Ceramide and S1P have been identified as bioactive signaling molecules that regulate cell growth, differentiation, senescence, apoptosis, and autophagy [40].

Numerous studies have suggested sphingolipids (SLs), as well as ceramide, are autophagosome membrane components [41, 42]. In recent years, the role of ceramide in autophagy has become known. The compound ceramide inhibits the transport of nutrients and initiates autophagy [15]. Ceramide stimulates autophagy by regulating classic or atypical autophagic pathways. By virtue of these signals, class I PI3Ks and AKT negatively regulate autophagy, whereas class III PI3Ks stimulate autophagy. Ceramide was determined to act as an inhibitor of AKT, by inducing phosphoprotein phosphatase 2A, and to promote interaction between PI3K class III and other autophagy regulators [43, 44]. Studies have shown that ceramide interacts directly with microtubule-associated protein light chain 3 (LC3) on mitochondrial membranes to induce deadly autophagy via an increase in intracellular mitophagy [45]. It has been reported that ceramide-induced autophagy can result in cell death under high growth factor conditions [15]. In addition, ceramides activate the transcription factor c-Jun, which is involved in the increase of the autophagic protein Beclin-1 [46]. The chemotherapeutic agents may induce the biosynthesis of intracellular ceramide that can result in autophagy-mediated cell death; however, an increase in S1P levels results from nutrition starvation and intercedes cytoprotective autophagy. Therefore, during the conversion of ceramide into S1P as a result of overexpression of acid ceramidase, the survival effects accumulate as well as the death signals are removed and it is suggested that acid ceramidase can influence the response to chemotherapeutics [46, 47].

Autophagy may support cellular survival and help cancer cells to resist metabolic and therapeutic stress [48]. Three types of autophagy are identified so far: macroautophagy (also called 'autophagy'), microautophagy, and chaperone-mediated autophagy (CMA) [45, 49]. During macroautophagy (autophagy), a double membrane structure called a phagophore engulfs intracellular components such as proteins and organelles. An autophagosome is formed when this transient compartment matures. Autophagosomes and lysosomes fuse to form autolysosomes, which digest vesicle contents for recycling. Macroautophagy has been linked to the development of AML in various studies. There is a correlation between the high expression of key genes involved in autophagic processes such as SIRT1 (sirtuin 1), BECN1 (beclin 1), STK11/LKB1 (serine/threonine kinase 11), and ATG7 (autophagy-related 7) and poor clinical outcome, short remission duration and drug resistance in patients with AML [50, 51].

Cancers such as prostate cancer, liver cancer, melanoma, and AML exhibit high expression of acid ceramidase [12, 52, 53]. Cancer cells expressing a high level of acid ceramidase may resist chemotherapy and radiation therapy since acid ceramidase inhibits apoptosis caused by TNF-α [45, 46, 54]. It is worth noting, that AC is not only found in lysosomes in cancer cells but is also present in the cytoplasm [46]. In a study by Turner et al. prostate cancer cell lines overexpressing acid ceramidase showed an increased lysosomal density and an increased autophagic activity [54]. In melanoma cells treated with doxorubicin, Michele Lai et al. found that acid ceramidase controlled apoptosis and increased autophagy [55]. Su-Fern Tan et al. demonstrated for the first time how targeting acid ceramidase reverses the aberrant lipid profile of AML, characterized by low ceramide levels and elevated S1P, which ultimately leads to cell death [12]. According to collected evidence, the upregulation of acid ceramidase in cancers can boost cytoprotective autophagy, which is associated with drug resistance in many types of cancers, especially AML.

## Sphingolipid metabolism

Sphingolipids are a large group of lipids that varies structurally from other lipids in that they have a sphingoid group as their structural backbone. Sphingolipid metabolism is a complex network in which the balance of sphingolipid formation, degradation, and recycling are carefully controlled in terms of cell response and fate.

Serine palmitoyl-transferase (SPT) catalyzes the condensation of serine and palmitoyl-CoA to produce 3-keto-dihydro sphinganine at the cytosolic side of the ER membrane, where de novo formation of sphingolipids occurs. The sphingoid base dihydrosphingosine (sphinganine) is generated from 3-keto-dihydro sphinganine, which, along with sphingosine, forms the backbone of sphingolipids.

Ceramide synthases (CERS1-6) catalyze the N-acylation of sphingoid bases, which leads to ceramide formation. The CERSs have variable chain length unique qualities [56], which adds to the sphingolipid's diversity. CERS1 favors C18-CoAs, whereas CERS2 uses acyl-CoAs in the range of C20 to C26. CERS3 has an affinity for ultra-long-chain acyl-CoAs (C26–36), whereas CERS4 has a preference for C18- and C20-CoAs. C16-CoAs are largely present in CERS5 and CERS6. Ceramides can be phosphorylated to produce ceramide-1-phosphate (C1P), modified with phosphocholine to generate sphingomyelin, or glycosylated to produce a wide range of glycosyl ceramides (Fig. 1).

Ceramides can be produced from sphingosine, recycled from glycosyl ceramides and sphingomyelin, and dephosphorylated from C1P [57]. Furthermore, while de novo ceramide formation by CERSs takes hours [58], ceramide generation through recycling pathways, such as sphingomyelin degradation, occurs within minutes after activation [59]. Therefore, ceramides and other sphingolipids potentially regulate cellular functions in the short term via salvaging pathways, while de novo sphingolipid synthesis might affect long-term cellular processes.

Given the critical roles of sphingolipids in autophagy, it is important to note that autophagy affects sphingolipid levels, particularly ceramide levels [60], and also the mobilization and storage of glycerolipids in lipid droplets [61–63]. Overall, research demonstrates that autophagy and lipid metabolism are coordinated, highlighting the relevance of sphingolipids in metabolic regulation. Some of the effects of sphingolipids have been documented, however, they are based on short-chain ceramides, which may have very different effects than ceramides generated in vivo. Instead of utilizing such non-natural ceramides, the involvement of sphingolipids in autophagy must be investigated further using genetically amenable model systems using loss-of-function and overexpression approaches.

## The role of autophagy in AML chemo-resistance

For decades, the use of chemotherapy to control and treat many cancers, such as AML has been common. However, this method has been along with several challenges, such as chemotherapy resistance. Importantly, autophagy is one of the most important mechanisms in chemotherapy resistance. A variety of studies revealed that multiple signaling pathways are related to energy metabolism and gene mutations can lead to autophagy [64]. Interestingly, reactive oxygen species (ROS), causes autophagy in AML cells via HIF/BNIP3/Bcl-2 [65]. It is noticeable to state that the level of ROS is increased in chemotherapy [66]. Sometimes it is possible that environmental conditions cause increase levels of ROS that are named stress signaling in general, such as the low level of oxygen, growth factors deprivation, acid amine deprivation, the low level of ATP, and lack of glucose [67]. In addition, temozolomide as a drug used in AML initiates autophagy and blockade of the apoptotic pathway via ERK/ROS signaling pathway [6]. To place more emphasis on cellular stress signaling, it is noticeable that these signaling lead to activation of JNK as a signaling factor and JNK causes activation of C-Jun, C-Fos, and FOXO1/3 as transcription factors and leads to an increased level of transcription of some autophagy factors (i.g Beclin 1, VPS34, and LC3) [65]. Also, stress pressure on AML cells activates heat shock transcription factor 1 (HSTF1) that launches transcription of ATG7 as an autophagy factor and chemo-resistance inducer [6]. Interestingly, AML treatment with some common drugs like Ara-C can induce autophagy and lead to drug resistance in AML cells. For instance, according to X Hu et al.'s studies, AML treatment with Ara-C leads to an increase expression of CXCR4 on AML cells. In this study, authors have found that CXCR4/SDF-1α axis can induce upregulation of SIRT1 in these cell lines [68]. Importantly, SIRT1 stimulates autophagy via deacetylation of ATG5, ATG7, ATG8, and LC3 [69]. On the other hand, Ara-C modulates several signaling pathways, such as AKT and ERK that lead to the activation of autophagy via mTOR pathway [70]. With this in mind, in addition to chemotherapy, recent treatments such as epigenetic therapies are no exception. Circular RNAs (circRNAs), are several types of non-coding RNAs used in some cancers, like AML. Accordingly, J. shang et al. have found that treatment with circRNAs in AML cells is associated with increased circPAN3 that induces autophagy through the AMPK/mTOR signaling pathway [17]. In a study by Jin Shang et al. [17] it has been demonstrated that circPAN3 may increase AML drug resistance by inducing autophagy. Also, Xiaojia Hu et al. [68] have found that the autophagy-related protein SIRT1 expression is associated with stimulation of SDF-1a-CXCR4 signaling, interacting with autophagy proteins (e.g. ATG5 and LC3). In addition, they have revealed that among primary human AML samples, a high CXCR4 expression level is correlated with SIRT1 and other autophagy-related proteins' elevated expression levels. Overall, their investigation proposed new roles for SDF-1a-CXCR4 signaling in the induction of autophagy in AML cells, which consequently facilitated their survival and drug resistance under stress. Specifically, in thisreview, we described that acid ceramidase and FLT3-ITD mutations can lead to autophagy in AML patients. In addition, multiple studies have revealed autophagy as a major cause of drug resistance in AML patients and other malignancies [17]. Therefore, acid ceramidase overexpression and FLT3-ITD mutation can increase drug resistance in these patients.

### The role of FLT3-ITD mutation in AML drug resistance

Midostaurin is a multi-targeted tyrosine kinase inhibitor that inhibits FLT3 [71]. The presence of FLT3-ITD and FLT3-TKD mutations in the same subclone of an AML patient may induce primary resistance to FLT3 inhibitors [72]. Because of the wide range of FLT3-ITD mutations, the position and amino acid sequence of ITD may also be involved in primary resistance by modifying protein structure, which activates alternate downstream signaling pathways [73]. It is unclear how mutant FLT3-internal tandem duplication (ITD) regulates cellular signaling pathways that contribute to AML cell death resistance. However, studies showed that FLT3-ITD targeting increased ceramide accumulation on outer mitochondrial membranes, which in turn resulted in the binding of autophagy-inducing light chain 3 (LC3), which is involved its I35 and F52 residues, to recruit autophagosomes to execute lethal mitophagy [74].

Quizartinib is a small molecule inhibitor with activity against FLT3-ITD. It is a very efficient FLT3 inhibitor that was designed specifically for this purpose. Unfortunately, when AML patients are treated with quizartinib, resistance develops fast, typically owing to secondary mutations in the FLT3 gene. Pexidartinib has been proven to be effective against FLT3-ITD, but the tumor is also resistant to pexidartinib due to additional secondary mutations [71, 75, 76]. Sorafenib (Nexavar) is a small molecule multitargeted kinase inhibitor that targets FLT3 [71]. Smith et al. identified secondary point FLT3 mutations in eight relapsed patients with ITD mutated AML. As well, these mutations have shown resistance to sorafenib [75].

Mutation in FLT3 is associated with poor prognosis in AML patients. Interestingly, mutations in FLT3 causes a kind of drug resistance. As part of a study on this issue, Dany et al. have found that the cause of drug resistance in FLT3 mutant AML cells is FLT3-ITD signaling that leads to the reduction of C18-ceramide. In this regard, CerS1 activation is stopped and we have stopped producing C18-ceramide [74]. On the other hand, when the activity of FLT3-ITD is suppressed with some drugs such as ASP2215, quizortinib, sorafenib, and cerenolanib, the percentage of cancerous cells death increases through mitophagy resulting from CerS1 activity that causes an increased level of C18-ceramide [10, 11]. Importantly, this mutation causes resistance to some drugs like Doxorabicin that target mitochondria via mitophagy, leading to AML cell death [12]. Furthermore, according to studies, mutation in FLT3 causes Ara-C resistance in AML cells due to a decreased uptake of Ara-C. To that end, the expression level of Ara-C transport which is named equilibrative nucleoside transport 1 (ENT1) is decreased fallowing HIF-1α activity [13]. According to Jin et al. increased HIF-1α activity originated from PI3K/AKT and MAPK/ERK signaling pathways that are activated directly by FLT3 [14]. Interestingly, increased activity of HIF-1α causes decreased expression of ENT1 [15].

### The role of acid ceramidase overexpression in AML drug resistance

Acid ceramidase inhibition has also significantly improved survival in mice with AML. In AML, acid ceramidase plays a crucial role in blast survival and drug resistance [77].

Bcl-2 is a crucial factor in the apoptosis signaling pathways. Researchers find that Bcl-2 inhibition overcomes apoptosis resistance and significantly increases survival in models of resistant AML [78]. In addition, it was shown that acid ceramidase activity promotes the production of pro-survival Mcl-1 protein and confers resistance to Bcl-2 inhibition [12].

The combination of daunorubicin (dnr) and cytarabine (Ara-C) is a critical component of AML treatment and resistance to these drugs greatly contributes to the failure of treatments [79]. Using parental HL-60 cells and drug-resistant derivatives as the model, researchers found that acid ceramidase overexpression in HL-60 induced resistance to the AML chemotherapy drugs such as cytarabine, mitoxantrone, and daunorubicin [80]. Turner et al. [81] have concluded that acid ceramidase overexpression enhances autophagy in prostate cancer and that enhances autophagy increases resistance to ceramide. The findings imply that prostate cancer cells overexpressing acid ceramidase have a significantly higher amount of autophagy than parental cell lines, thus, resulting in an 'insult-ready' phenotype in which cells are more resistant to initial insult and can quickly metabolize any ceramide generated. Therefore, they believe that inhibition of autophagy improves treatment response. According to studies, acid ceramidase is significantly increased in AML blasts. Importantly, when sphingosine (SPH) is made by acid ceramidase, it is phosphorylated by sphingosine kinase (SPHK), and sphingosine 1 phosphate (S1P) levels rise in the AML blasts. This series of events causes upregulation of NF-κB (Fig. 1) [16]. NF-κB in AML blasts actives P-glycoprotein (P-gp)/multidrug resistance protein 1 (MDR1) promoter. Interestingly P-gp is a protein with a flexible structure that causes leaking out of the drugs that are used for AML treatment [17].

## Conclusion and future directions

Chemo-resistance as the major challenge in AML therapy, reduces the survival rate and treatment failure in multiple malignancies such as AML. In order to overcome this serious issue, the mechanisms of chemo-resistance in AML must be uncovered. One of the mechanisms of chemo-resistance in AML is autophagy. In this study, we suggested that FLT3-ITD and high levels of acid ceramidase could lead to chemo-resistance by inducing autophagy. In patients with AML, some types of gene mutations such as FLT3-ITD, overexpression of some chemo-resistance-related enzymes such as acid ceramidase, and autophagy signaling pathways, lead to relapse and chemo-resistance. By targeting these adverse factors, chemo-resistance could be alleviated. Patients with AML must be evaluated to determine whether they harbor high-risk factors for drug resistance, as we suggested in this study FLT3-ITD and overexpression of acid ceramidase could be considered as adverse factors that lead to autophagy and chemo-resistance in these patients. Therefore, the combination of drugs targeting FLT3-ITD and its downstream signaling pathways, acid ceramidase, and autophagy with common chemotherapies might be a promising therapeutic approach for these AML patients in order to overcome chemo-resistance and improve long-term survival rate. However, further investigations are recommended to evaluate the interplay between FLT3-ITD and acid ceramidase in acute myeloid leukemia chemo-resistance and subsequently, more clinical, in-vitro, and in-vivo studies are needed to better understand these mechanisms and prepare therapeutic strategies for them.

## Data Availability

Not applicable.

## Abbreviations

AML: Acute myeloid leukemia
FLT3-ITD: Fms-like tyrosine kinase 3-internal tandem duplication
ATG: Autophagy-related gene
Ara-C: Cytarabine or cytosine arabinoside
MDR: Multidrug resistance
HIF-1α: Hypoxia-inducible factor-1 alpha
ROS: Reactive oxygen species
NF-κB: Nuclear factor kappa B
PI3K: Phosphatidylinositol-3-kinase
mTOR: Mammalian target of rapamycin
MAPK: Mitogen-activated protein kinase
ERK: Extracellular signal-regulated kinase
Cer: Ceramide
Sph: Sphingosine
dHCer: Dihydroceramide
dHSph: Dihydrosphingosine
C1P: Ceramide-1-phosphate
S1P: Sphingosine-1-phosphate
3KSR: 3-Ketosphinganine reductase
CPP: Ceramide phosphatase
DES1: Dihydroceramide desaturase 1
aCDase: Acid ceramidase
nCDase: Neutral ceramidase
SPHK: Sphingosine kinase
aSMase: Acid sphingomyelinase
SPL: S1P lyase
S1PP: Sphingosine phosphate phosphatase
